# Comparison of clinical presentation and treatment response among youth with atypical anorexia nervosa and anorexia nervosa

**DOI:** 10.1371/journal.pone.0316572

**Published:** 2025-03-04

**Authors:** Bek Urban, Kelly Cai, David M. Freestone, Megan Hellner, Cara Bohon, Adam Arsenault, Dori M. Steinberg, Jessica H. Baker

**Affiliations:** 1 Equip Health, Inc., Carlsbad, California, USA; 2 School of Community Health Sciences, Counseling, and Counseling Psychology, College of Education and Human Sciences, Oklahoma State University, Stillwater, Oklahoma, USA; 3 Department of Psychiatry and Behavioral Sciences, Stanford University, Stanford, California, USA; 4 School of Nursing, Duke University, Durham, North Carolina, USA; UClan, UNITED KINGDOM OF GREAT BRITAIN AND NORTHERN IRELAND

## Abstract

**Objective:**

Atypical anorexia nervosa (AAN) is an eating disorder that shares all symptoms with anorexia nervosa (AN) except “significantly low weight.” Here, we aim to further understand the potential similarities and differences between AN and AAN in a clinical sample of patients receiving family-based treatment (FBT) for AN or AAN. The objectives of this study were to 1) compare clinical presentations among adolescent and young adult patients with AN and AAN; and (2) evaluate and compare treatment response to FBT among these patients.

**Method:**

Clinical data from 1,438 patients (M = 15.3 years, SD = 2.51) undergoing virtual augmented family-based treatment (FBT) who met research specified criteria for AN or AAN were analyzed. To provide the most robust comparison, four common definitions of AAN identified in previous research were retroactively applied to create comparison groups and assess possible differences in symptom presentation and treatment response.

**Results:**

The percentage of patients classified as AAN ranged from 20%–81.3% depending on definition. Patients with AAN presented with slightly higher eating disorder and depression symptomatology in half of the definitions. All AAN groups gained weight more slowly throughout treatment; other symptom improvement was similar between groups.

**Conclusions:**

Results show more similarities than differences in presentation and treatment response among patients with AN and AAN and confirm previous pilot studies showing FBT is effective for AAN. Although currently diagnostically distinct, apparent differences between AN and AAN were not found in this study, suggesting a re-consideration of this diagnostic separation.

## Introduction

Atypical anorexia nervosa (AAN) is a restrictive eating disorder within the other specified feeding or eating disorder (OSFED) diagnosis that was first included in the 5th edition of the Diagnostic and Statistical Manual of Mental Disorders (DSM-5).[1] Like anorexia nervosa (AN), patients with AAN display symptoms of: 1) restriction of energy intake relative to requirements, 2) significant weight loss, 3) an intense fear of gaining weight or persistent behavior that interferes with weight gain, and 4) disturbance in the way in which one’s body weight or shape is experienced, undue influence of body shape/weight on self-evaluation, and/or persistent lack of recognition of seriousness of disorder.[2] Unlike AN, however, individuals with AAN do not have “significantly low body weight” and maintain a body weight “within or above normal range,” despite energy restriction and significant weight loss. It is important to note that significant ambiguity exists around the diagnostic definition for significant weight loss and what constitutes “within or above normal range” to be diagnosed with AAN.[2] In a recent systematic review, 75 separate studies of AAN used 29 unique diagnostic operationalizations of AAN.[3] Although prevalence data for AAN is sparse, AAN appears to have a lifetime prevalence three to four times greater than AN, making it vital that we have a clear definition that allows us to better understand AAN and how to treat it.[3]

Despite having distinct diagnostic classifications, there are few clinically-relevant differences between patients with AN and AAN,[4] such that machine learning models fail to differentiate individuals with AN from those with AAN based on factors other than body mass index (BMI).[5] Indeed, many negative physical health impacts (e.g., bradycardia, GI complications, neurological impacts),[6–8] degree of caloric and food group restriction,[9] rates of adolescents hospitalized for medical stabilization, and level of care required to establish medical stability [6,10] are similar across the two diagnoses. Notably, some studies have also shown *more severe* psychopathology among those with AAN,[11] which may be due to experiences of weight stigma and bullying,[12,13] delayed access to care,[12] and/or praise for weight loss.[12] Some studies have reported minor differences between the diagnoses in relation to bone density and blood pressure. [6,14]

Family based treatment (FBT) is generally the first line treatment recommended for adolescents with AN.[15,16] FBT incorporates family members into the treatment team along with a trained psychologist or therapist, medical provider, and a dietitian.[15] It is the caregivers’ responsibility to guide and supervise at-home aspects of treatment during the early stages of treatment. For example, a primary role of caregivers is to supervise and monitor meals and eating disorder behaviors, which supports the child’s recovery.[15] FBT has resulted in better remission outcomes than comparison treatments for adolescents with AN [16–19 ] and preliminary work suggests it may be effective for AAN.[20–22] Indeed, in a case series of FBT for AAN, 37.5% of AAN patients achieved full remission and an additional 25% achieved partial remission.[22] However, to our knowledge, the effectiveness of FBT for AAN has not been evaluated in large, community-based patient samples nor directly compared to AN.

Taken together, previous research suggests that there may be more similarities than differences between AN and AAN and calls into question the need for separate diagnostic classifications, particularly given the lack of an agreed upon definition of significant weight loss and “normal” weight for AAN. Here, we aim to further understand the similarities and potential differences between AN and AAN in a clinical sample of patients receiving FBT for AN or AAN. The aims of this study are to: (1) evaluate and compare the clinical presentation among adolescent and young adult patients with AN and AAN; and (2) evaluate and compare treatment response to FBT among patients with AN and AAN using several research-defined diagnostic definitions for AAN.(3) Evaluating multiple definitions will provide the most robust comparison of patients with AN and AAN possible and ensure that comparisons are not dependent on specific definitions of AAN.

## Method

### Participants

Participants were identified through a retrospective chart review of patients receiving intentionally virtual eating disorder treatment (i.e., treatment designed to be delivered virtually) from September 2020 to July 31st, 2023, when data was accessed for research purposes. Included patients were those whose charts indicated they were diagnosed with AN or other specified feeding or eating disorder-AAN following a semi-structured interview at treatment intake with a clinician. Patient presentation and treatment response data was obtained and collected during the course of treatment. Evaluation of our existing treatments and outcomes were reviewed by the Western Institutional Review Board, and was deemed exempt from IRB oversight. Additionally, at treatment admission, patients (or caregivers of minors) provided informed consent for treatment data to be evaluated and published for research purposes. Although the patient medical records from which the data for this study were taken contain identifying patient information, with the exception of medical record number, data was extracted from the record without identifying information.

### Treatment overview

Patients received care at a virtual eating disorder treatment program, using an augmented family-based treatment (FBT) modality. The augmented FBT modality included a treatment team composed of a therapist, registered dietitian, medical provider, a peer mentor, and a family mentor. Mentors are unique members of the augmented FBT approach and not part of a ‘standard’ FBT treatment team. Mentors are individuals who have lived experience recovering from an eating disorder (peer mentor) or caring for an individual recovering from an eating disorder (family mentor). More detailed information about the treatment model and effectiveness has been published elsewhere.[23,24]

### Measures

Patients and/or families completed validated survey measures on either a weekly or monthly basis during treatment in order to monitor treatment progress. All measures are completed as part of standard care. Measures were completed via a HIPAA-compliant telehealth platform.

#### Height and weight.

Patient weight was recorded at home, collected by a family member trained on weighing their child during treatment orientation or by a patient’s primary care team. Height was reported at intake and used to calculate patient body mass index (BMI) at admission. For patients under 18, BMI percentile was determined using the Center for Disease Control’s age-adjusted BMI growth charts for cisgender girls and boys.[25] We used sex assigned at birth to determine which curve to use for each patient, as BMI reference charts are unavailable for transgender and gender diverse youth.

The target weight, or expected body weight (EBW), for each patient was determined by the patients’ registered dietitian using an individualized approach that relies on the patient’s historical medical records to track their individual growth pattern and trajectory. Although weight restoration guidelines for patients with AAN have not been established, weight restoration plans for adolescents with FBT are common in practice [20] and a need for patients with AAN to return to their premorbid weight has been well supported.[26] Thus, although patients with AAN are not classified as underweight, there may be a need for weight restoration. According to this approach, a patient’s EBW was set at a target that would return patients to their pre-eating disorder weight or growth curve trajectory, independent of diagnosis.

#### Defining AN and AAN.

Because ambiguity exists around the diagnostic definition for significant weight loss and what constitutes “within or above normal range” to be diagnosed with AAN [2,3], we define AAN diagnosis according to the four most common research definitions used in previous studies.[3] This will provide additional confidence that the results obtained here are not simply dependent on specific definitions of AAN being used. Through a chart review, we retrospectively classified patients as AN or AAN according to one of the four AAN definitions described below. Using these four definitions allows us to understand what our sample would look like if a differing AAN definitions were used.

Because BMI is the only differentiating feature between AN and AAN, we tested definitions that used varying BMI cutoffs to differentiate AN and AAN. Patients who did not meet one of the AAN classifications below and otherwise met AN criteria were included in the AN group. Patients could be classified as AN in one definition and AAN in another.

1. **AAN-DSM5**: BMI at admission greater than or equal to 19 kg/m^2^ for adults aged 18 or older, or above the median BMI for patients under 18.
2. **AAN-BMI-18.5:** Admission BMI greater than or equal to 18.5 kg/m^2^ for adults or greater than or equal to 85% of the median BMI for adolescents and children.
3. **AAN-BMI-10th Percentile:** Admission BMI over the 10th percentile–only relevant for children/adolescents.
4. **AAN-DSM5 HLOC:** Admission BMI was 19 kg/m^2^ or above for adults or above the median for adolescents, and the patient had not previously received treatment at a higher level of care (HLOC; e.g., residential treatment, inpatient). Here, the BMI thresholds are the same as the DSM-5 definition. However, patients with previous eating disorder treatment at a HLOC were classified as AN even if their BMI was above the threshold. The rationale for this definition [27] is that previous HLOC treatment may have resulted in weight gain placing an AN patient outside of the required BMI threshold for an AN diagnosis; however, this weight gain occurred within the course of AN and therefore, would not be a new diagnosis.

#### Surveys.

**Eating disorder symptom severity.** The Eating Disorder Examination-Questionnaire Short Form (EDE-QS) [28] is a 12-item self-administered survey adapted from the Eating Disorder Examination Questionnaire.[29] The EDE-QS was administered weekly and measures the frequency of eating disorder behaviors and thoughts in the past seven days on a four-point scale ranging from zero (no days) to three (six to seven days). Higher scores indicate more severe psychopathology.

**Depression.** Depression was assessed monthly using the nine-item Patient Health Questionnaire [30] (PHQ-9). The PHQ-9’s validity for capturing depression across a wide array of populations is widely supported. Patients indicate how frequently they have experienced a symptom over the last fourteen days from 0 (not at all) to 3 (nearly every day). Higher scores indicate greater symptomatology.

**Anxiety**. Patients completed the Generalized Anxiety Disorder Questionnaire [31] (GAD-7) monthly to measure the frequency of anxiety-related symptomatology in the preceding fourteen days. Patients select from response options spanning from zero (not at all) to three (nearly every day), with higher scores reflecting greater symptomatology.

**Caregiver burden**. Caregivers completed the 19-item self-report Burden Assessment Scale [32] (BAS) each month to evaluate caregiver burden related to the eating disorder. The questionnaire measures impacts caregivers may experience such as financial problems, family friction, and guilt. Caregivers respond to items using a four-point Likert scale from one (indicating no burden at all) to four (indicating a significant amount of burden) as a measure of overall felt burden, with higher scores indicating a more pronounced sense of perceived and actual caregiving burden.

### Statistical analysis

Descriptive analyses were used to characterize the presentation of the sample at admission. Kolmogorov-Smirnov tests and chi-squared tests were used to detect statistical differences among demographic variables across the groups. Holm-Bonferroni corrections were used to maintain a family-wise alpha of 0.05 for significance testing.

Differences in clinical presentation at admission and throughout treatment between patients classified as AN and AAN were estimated using linear mixed effects models. For each definition, we ran separate linear models for each outcome measure described above (20 models total). All models followed from the same underlying linear model, and included terms for the patient’s classification based on each of the four AAN definitions, the log of treatment week, and their interaction. Age at admission was used as a covariate. Using Wilkinson’s notation, the models took the form: y ~  1 +  admission_age +  classification*log(treatment_week) +  (1 +  log(treatment_week) |  patient). AN patients were the reference group. Analyses were performed in R version 4.3.1. Fitting was done using lmerTest package [33] version 3.1–3. The targets package was used for project management (version 1.2.0 [34]).

## Results

### Patient characteristics

**Fig 1:**
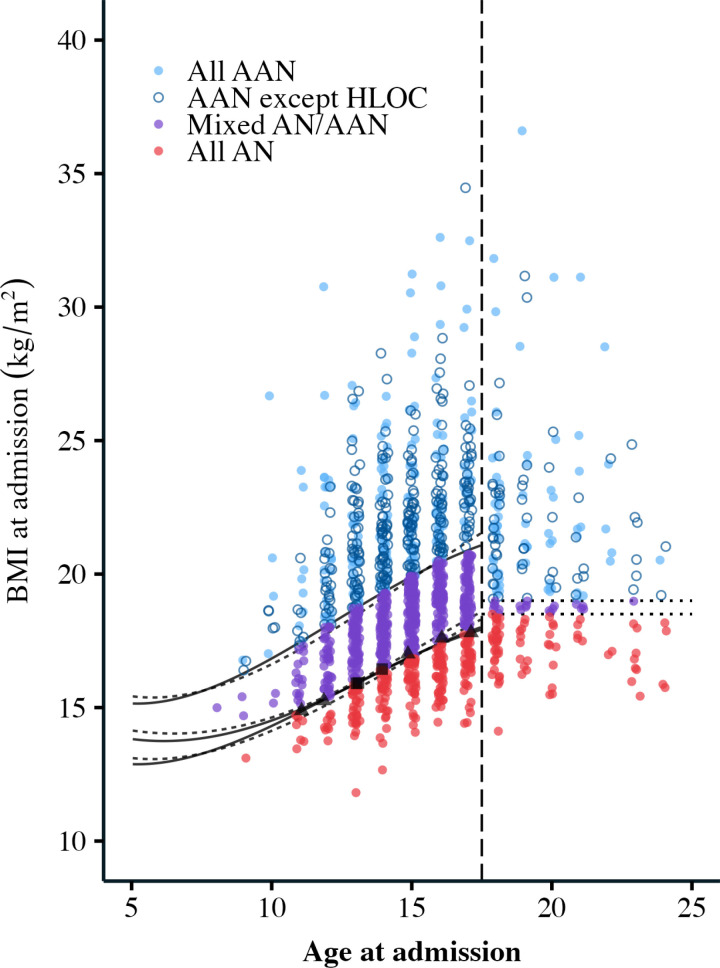
Patient classifications under each definition. Each point represents one patient. Age at admission is plotted on the x-axis, and BMI at admission on the y-axis. The vertical dashed line separates adults (18 and over) and children/adolescents. The solid and dashed curves on the left side represent the 50th, 10th, and 85% of the 50th percentile for females and males respectively, based on CDC growth charts. The dotted lines on the right side represent BMIs of 19 and 18.5 kg/m^2^. The red patients at the bottom (n = 288) were classified as AN under all four definitions. The blue closed circle patients at the top (n = 297) were classified as AAN under all four definitions. The blue open circle patients at the top (n = 326) were classified as AAN under all definitions except the DSM5 HLOC definition – any patient who had prior HLOC treatment was classified as AN in this definition, regardless of their BMI. The purple patients in the middle (n = 519) were classified as AN in the DSM5 and DSM5 HLOC definitions, and as AAN in the BMI-18.5 and BMI-10th definitions. Since the thresholds for being classified as AAN were lower for BMI-18.5 and BMI-10th, these patients moved from AN to AAN. The thresholds for BMI-18.5 and BMI-10th were almost the same value for most patients, but in a few cases, a patient was classified as AAN in  BMI-18.5 and AN in BMI-10th (n = 5 patients in black, triangle), or AN in BMI-18.5 and AAN in BMI-10th (n = 3 patients in black, square). These points lie along the bottom set of curves. The figure excludes 4 patients who had a BMI at admission above 40 kg/m^2^ in order to increase visibility, but all statistical analysis includes them. Points are slightly jittered along the x-axis to increase visibility.

The initial sample included N = 1,660 patients. Due to missing data required to complete the analyses (e.g., height, sex assigned at birth), the final sample for analysis included 1,438 patients. Patients ranged from 8 to 25 years old (M =  15.3, SD =  2.51) and were primarily white (n =  1009, 70.2%) and cisgender girls/women (n =  1230, 85.5%). The number of patients classified into the AN and AAN analysis groups varied greatly depending on which definition of AAN was used (Table 1). The percentage of patients classified as AAN ranged from 20% to 81.3%. Some patients were classified as AAN under all four definitions (n =  288), some were classified as AN under all four definitions (n =  297), but a majority changed diagnosis across the various AAN definitions (n =  853) (Fig 1).

**Table 1:** AN and AAN classifications.

	Definition of AAN	AN Count (%)	AAN Count (%)
DSM5	Admission BMI ≥ 19 kg/m^2^ for adults; > median BMI for adolescents and children	824 (57.3%)	614 (42.7%)
BMI-18.5	Admission BMI ≥ 18.5 kg/m^2^ for adults; ≥ 85% of median BMI for adolescents and children	300 (20.9%)	1138 (79.1%)
BMI-10th	Admission BMI >10th percentile for adolescents and children	227 (18.7%)	990 (81.3%)
DSM5-HLOC	Admission BMI ≥ 19 kg/m^2^ for adults; > median BMI for adolescents and children **AND** no previous eating disorder treatment at a higher level of care than outpatient	1150 (80%)	288 (20%)

Note: The table displays counts and percentages of patients classified as AN and AAN under each definition.

### Clinical presentation at admission

Eating disorder symptoms at admission are shown in Table 2. For all definitions, patients who were classified as AAN had higher weights at admission than patients who were classified as AN (p <  0.001 in all cases). The proportion of patients classified as AN who required weight restoration ranged from 85.1% to 94% (depending on the AAN definition used), while the proportion of patients classified as AAN who required weight restoration ranged from 71.5% to 81.4%. Patients classified as AN were significantly more likely to require weight restoration than AAN patients under all definitions (all p <  0.001). The median weight gain needed to reach EBW at admission for AN patients ranged from 22.1 to 27.9 pounds depending on the definition used, which was significantly more than the 17.2 to 19.6 pounds of weight to reach EBW among AAN patients (all p <  0.001). Eating disorder symptoms were slightly higher (approximately 2 point difference on EDE-Q) for AAN patients than for AN patients in two of the four definitions (DSM5 and DSM HLOC).

**Table 2:** Differences in eating disorder symptom severity at admission and week 16 of treatment.

	Symptoms at admission		Symptoms at week 16	
	AN	AAN	AN	AAN
Weight				
* DSM5*	98.9 (0.6)	**121.9 (0.8)**	113.3 (0.6)	**131.2 (0.8)**
* BMI-18.5*	89.6 (1.0)	**113.0 (0.6)**	104.8 (1.1)	**124.6 (0.6)**
* BMI-10th*	86.5 (1.1)	**110.9 (0.6)**	102.5 (1.2)	**122.8 (0.6)**
* DSM5 HLOC*	104.3 (0.6)	**121.6 (1.3)**	117.3 (0.6)	**131.5 (1.3)**
EDE-QS				
* DSM5*	13.7 (0.3)	**15.4 (0.4)**	6.4 (0.3)	8.4 (0.4)
* BMI-18.5*	12.9 (0.6)	14.8 (0.3)	6.5 (0.5)	7.4 (0.3)
* BMI-10th*	13.0 (0.7)	14.7 (0.3)	6.4 (0.6)	7.4 (0.3)
* DSM5 HLOC*	13.7 (0.3)	**17.2 (0.6)**	7.0 (0.3)	**8.2 (0.5)**

Note: One model was run for each combination of definition and symptom measure (20 models total, GAD-7, PHQ-9, and carer burden are not shown, see text for results). The first two columns (symptoms at admission) show the model estimates for each symptom measure at admission for AN and AAN, with standard errors in parentheses. Estimates are based on the mean patient age (15.3 for DSM5, BMI-18.5, and DSM5 HLOC, and 14.5 for BMI-10th). Cells are bolded to indicate significant differences between the model intercepts for AN and AAN after Holm-Bonferroni correction. The last 2 columns (symptoms at week 16) show the model estimates at week 16 of treatment, with standard errors in parentheses. Cells are bolded to indicate significant differences between rates of change over time for AN and AAN after Holm-Bonferroni correction. Model estimates are shown instead of raw means in order to account for missing data in specific weeks and differences based on age.

For associated symptoms evaluated at admission, depression scores were slightly higher for two AAN groups compared with the AN group (DSM5: AN 9.1 (0.3), AAN 10.6 (0.3), t =  3.6, p <  0.001; DSM HLOC: AN 9.4 (0.2), AAN 11.1 (0.4), t =  3.4, p <  0.001); however, this difference is unlikely to be clinically meaningful (approximately 1 point difference on PHQ-9). Finally, caregiver burden was significantly lower for patients in the DSM5 HLOC AAN group than for patients classified as AN in one case (approximately 2 point difference on BAS; AN 44.2 (0.3), AAN 42.2 (0.6), t =  ‒3.1, p =  0.0023). All other comparisons were non-significant.

Taken together, results indicate that the clinical presentation of AN and AAN to treatment is quite similar. Few marked differences existed and those that did are unlikely to be clinically meaningful. While more than half of AN and AAN patients required weight restoration, AN patients had more weight to gain to reach EBW and were significantly more likely to be on a weight restoration plan. This is unsurprising given the core difference between AN and AAN is weight/BMI.

### Treatment outcome

Overall, there were few significant differences in the trajectory of treatment outcomes for AN or AAN patients for any measure (Table 2). Both groups, regardless of the AAN definition used, improved throughout treatment at similar rates. There were two exceptions. First, in all cases, weight gain progressed more slowly for AAN patients than AN patients. Second, patients classified as AAN using the DSM5-HLOC definition showed slightly faster eating disorder symptom improvement, about a 2-point difference by 20 weeks. However, we do not consider this to be clinically significant.

### Post-hoc analysis

In the sections above, we segmented the analysis sample into discrete categories of AN and AAN according to common thresholds used in the literature. Here, rather than creating distinct categories, we completed a post-hoc analysis to directly evaluate if an association exists between BMI and symptoms scores. When BMI was treated as a continuous variable, weight was the only outcome that showed significant differences, such that patients with higher BMIs at admission had higher weights at admission and gained weight at a slower rate over time (all p <  0.001). There was no association between BMI and the other symptom measures.

## Discussion

We compared the clinical presentation and treatment outcomes in a large sample of patients receiving FBT. Despite testing four definitions of AAN that have been most widely used in previous research,(3) our results did not show clinically distinct differences in patients with AN and AAN in clinical presentation or treatment response to FBT. In general, findings were consistent across AAN definitions, indicating there are very few clinically significant differences between patients classified as AN and AAN. Lack of difference does not depend on the particular classification used, making it more robust and reliable. Further affirming these findings, we also saw no associations between BMI and symptom scores.

Notably, as described above, there is no established definition for “significant weight loss” or “normal weight” for an AAN diagnosis. Thus, over 25 unique diagnostic operationalizations of AAN have been used across 75 studies.(3) The breadth of definitions used makes it difficult to compare results across studies. Indeed, when applying the four definitions of AAN used here, individual diagnoses applied to patients did not remain consistent: 59% of patients were classified as AN at least once and AAN at least once. This high level of variability emphasizes the importance of standardized definitions for diagnostic criteria.

Findings here did show significant weight and BMI differences between AN and AAN such that the average weight and BMI for AAN groups were higher than AN groups. This is unsurprising given that the core (and only) diagnostic difference between AN and AAN is BMI. A critical component of FBT for AN is weight restoration and while patients with AN were significantly more likely to be on a weight restoration plan, approximately 75% of patients with AAN were identified by dietitians as requiring weight restoration. Depending on the definition used, patients classified with AN required 22.1 to 27.9 pounds on average of weight gain to reach EBW whereas patients classified with AAN required 17.2 to 19.6 pounds on average. Although patients classified with AN also needed to gain significantly more weight to reach EBW than patients with AAN, patients classified with AAN still needed to gain a non-trivial amount of weight to restore weight to their premorbid eating disorder growth trajectory.

Returning patients to their pre-eating disorder growth trajectory is an important consideration given the impact weight suppression (i.e., difference between highest weight and current weight) can have on treatment success. For example, a higher BMI at the end of treatment for patients with AN is associated with better 6- and 12- month outcomes [35] whereas weight suppression at one year follow-up was associated with a poorer prognosis for adolescents with AAN [36]. Similarly, adolescents with an eating disorder who were previously ≥ 85th percentile BMI required a similar amount of weight gain as eating disorder patients not previously ≥ 85th percentile BMI for return of menses. [37] Although initial weight loss may have been praised or encouraged in patients presenting with AAN, taking into account pre-morbid eating disorder growth trajectory is critical to determining weight restoration goals.

One treatment difference that did emerge between the AN and AAN groups was that patients classified with AAN gained weight at a slower rate. We can only hypothesize the reasons for this, but one potential explanation is that FBT requires parent/caregiver buy-in. Given patients with AAN often start at a higher premorbid weight [38] and are not underweight, parents/caregivers may be less concerned about their child’s weight loss. As such, providers may have to challenge initial resistance from caregivers, including working with caregivers to strengthen commitment to treatment, defusing weight stigma and challenging weight bias, and providing education on the seriousness of AAN.

Taken together, the results of this study indicate that there are more similarities than differences in the clinical presentation of AN and AAN and that FBT is similarly effective for the treatment of AAN as for AN. The only consistent symptom differences that were observed in this study were related to weight/BMI–which is the sole diagnostic difference between these two illnesses. Because the threshold at which AN and AAN are separated is largely atheoretical, as evidenced by the plethora of definitions that have been used in the literature,[3] we tested various definitions of AAN as well as BMI as a continuous variable to ensure results were not due to artificial categorization, strengthening the reliability of these findings. As such, use of a single demographic difference may not be a strong basis of diagnostic separation. Weight status may provide meaningful information about an individual’s experience in the world and be associated with experiences of clinical focus, such as weight stigma, delayed access to care, and praise for weight loss associated with one’s eating disorder,[12,13,39–42] but may have limited utility diagnostically. Further, the diagnostic separation between AN and AAN can pose a hardship for patients with AAN in receiving coverage for treatment by insurance due to weight not being “low enough” and when care is received a lack of diagnostic clarity can create a challenge for clinicians in making an AAN diagnosis.[43–45] As such, we add to calls [4,5,11,46–48] for further consideration of the need for separating AN and AAN into distinct diagnoses or removing BMI/weight from AN diagnostic criteria entirely.

### Strengths and limitations

There are several notable strengths of this study. The large sample size presented here allowed for in-depth analyses and a robust approach to examining potential between group differences. Furthermore, this retrospective chart review in a naturalistic setting may be more representative of current clinical practice and more widely generalized. In contrast, due to the naturalistic setting, there was limited control over specific doses of treatment and variability across a patient’s treatment history (e.g., stepping down from HLOC, first time in treatment). We currently do not have post-treatment data in order to compare longer-term treatment outcomes.

## Conclusions

To provide the most robust comparison of patients with AN and AAN possible, we tested four common definitions of AAN used previously in research. In general, our findings suggest more similarities than differences between the clinical presentation and treatment outcomes for AN and AAN. Importantly, this study also provides additional support for FBT as the first line treatment for AAN as well as considering the need for weight restoration. However, clinicians should be aware that the initial stages of FBT could require a longer focus on parent buy in, commitment, and psychoeducation. Finally, the lack of an established definition for significant weight loss and normal weight poses a hardship for patients with AAN in receiving care despite similar consequences as AN. As such, we add to calls [4,5,11,46–48] for further consideration of the need for separating AN and AAN into distinct diagnoses or removing BMI/weight from AN diagnostic criteria entirely.
